# Benefits of using immersive virtual reality in haptic dental simulation for endodontic access cavity training: A comparative crossover study

**DOI:** 10.1111/iej.14252

**Published:** 2025-05-12

**Authors:** Octave N. Bandiaky, Valériane Loison, Christelle Volteau, Raphaëlle Crétin‐Pirolli, Sébastien George, Assem Soueidan, Laurent Le Guehennec

**Affiliations:** ^1^ Oniris, Univ Angers, CHU Nantes, INSERM, Regenerative Medicine and Skeleton Nantes University Nantes France; ^2^ Nantes Educational Research Center, CREN, Le Mans University Nantes France; ^3^ CHU Nantes, Research and Innovation Direction, Methodology and Biostatistics Platform Nantes University Nantes France; ^4^ Computer Science Laboratory of Le Mans University, Le Mans University Le Mans France; ^5^ Department of Periodontology, Faculty of Dental Surgery University of Nantes Nantes France; ^6^ Department of Prosthodontic, Faculty of Dental Surgery University of Nantes Nantes France

**Keywords:** cybersickness, dental education, endodontic access cavity, haptic simulation, immersive virtual reality, student perception

## Abstract

**Introduction:**

The use of haptic virtual reality simulators in preclinical dental education is evolving rapidly. However, the application of immersive haptic simulations for specific dental procedures, such as access cavity preparation, has not been extensively explored.

**Aims:**

This study aimed to (i) evaluate the impact of using the VirTeaSy Dental® simulator in conjunction with a virtual reality (VR) headset on student performance during access cavity preparation, with a focus on haptic parameters; (ii) assess students' perceptions of the experience; and (iii) examine the side effects associated with VR headset use.

**Methodology:**

The study included 90 third‐year dental students from the Dental Faculty of Nantes University, enrolled in January 2023. Participants were divided into two parallel groups. In Phase 1, Group 1 (*n* = 45) completed two endodontic access cavity exercises on the VirTeaSy Dental® without the VR headset, whilst Group 2 performed the same exercises using the VR headset. In Phase 2, the groups switched conditions and followed the same protocol. Performance was assessed using haptic parameters, and comparisons between groups for each phase were made using parametric and non‐parametric tests (*p* < .05). Students also completed questionnaires to assess their experience and report any side effects from using the VR headset.

**Results:**

Across both groups and phases, participants performed better in access cavity preparation without the VR headset. They showed greater accuracy, made fewer errors, and completed the exercises more quickly. Notably, more students failed to complete the exercises within the 10‐minute time limit when using the VR headset (27 vs. 12 in Group 1, 23 vs. 13 in Group 2). Most participants expressed a preference for using VirTeaSy Dental® without the VR headset. Approximately, 20% of students reported side effects, including dizziness, nausea, migraines, and neck muscle fatigue.

**Conclusion:**

The results suggest that full immersion in haptic simulation, when paired with a VR headset, negatively impacts student performance in complex tasks such as access cavity preparation. These findings underscore the current limitations of immersive virtual reality in dental education and highlight the need for technical refinements before its widespread adoption in preclinical training.

## INTRODUCTION

Preclinical dental education is undergoing a transformative shift with the increasing integration of virtual reality (VR) and haptic technologies in dental schools worldwide (Bandiaky, Loison, et al., 2024; Bandiaky, Lopez, et al., 2024; Hsu & Chang, 2023; Ibarra‐MartÃnez et al., 2024; Joseph et al., 2014; Moussa et al., 2022; Ranauta et al., 2025; Serrano et al., 2023; Vincent et al., 2020; Yamaguchi et al., 2013). These innovations aim to enhance students' acquisition of motor skills by providing immersive, interactive, and highly controlled learning environments (Buchanan, 2004; Corrêa et al., 2017; Daud et al., 2023; Farag & Hashem, 2021; Hattori et al., 2022; Mallikarjun et al., 2014; Patil et al., 2023; Perry et al., 2015; Pottle, 2019; Rodrigues et al., 2022; Suebnukarn et al., 2010; Ziane‐Casenave et al., 2022). Amongst these advancements, haptic virtual reality simulators (HVRS) offer unparalleled benefits, including unlimited, reproducible training opportunities (Baechle et al., 2022; Jasinevicius et al., 2004) real‐time feedback, and enhanced self‐assessment capabilities. By replicating complex clinical scenarios with precision, HVRS enable students to develop critical procedural skills with greater accuracy and confidence (Durham et al., 2019; Nassar & Tekian, 2020; Perry et al., 2015; Roy et al., 2017; Slaidina et al., 2025; Vincent et al., 2022; Zhao et al., 2020; Zorzal et al., 2021). The early integration of these technologies in preclinical dental training has been shown to significantly improve students' motor skills, self‐efficacy, and procedural precision, particularly in tasks such as milling and inferior alveolar block (IAB) anaesthesia (Collaço et al., 2021; Koo et al., 2015; Murbay et al., 2020; Urbankova, 2010).

A review of the literature reveals that most studies assessing the educational value of HVRS have been conducted without VR headsets, relying instead on non‐immersive environments (Bandiaky, Loison, et al., 2024; Bandiaky, Lopez, et al., 2024; Ben‐Gal et al., 2013; de Boer et al., 2016, 2017; Eve et al., 2014; Gal et al., 2011; Suebnukarn et al., 2014; Urbankova et al., 2013; Urbankova & Engebretson, 2011). Whilst these studies have demonstrated outcomes comparable to conventional simulators in developing the motor skills necessary for procedures such as endodontic access cavity preparation (Slaczka et al., 2024), there is a notable lack of data on the effectiveness of immersive VR combined with haptic technology (Bandiaky, Loison, et al., 2024; Bandiaky, Lopez, et al., 2024; Joda et al., 2019). Typically, these non‐immersive simulators display a 3D scene on a 2D screen, occasionally supplemented with passive or active 3D glasses. However, they do not provide a fully immersive experience. In contrast, immersive haptic simulation, facilitated by VR headsets, allows users to engage in operative procedures within a fully interactive virtual environment (Prendergast et al., 2009). Equipped with high‐resolution optical displays, VR headsets create an immersive world where students can realistically interact with virtual patients, dental instruments, and clinical scenarios. This level of immersion not only enhances spatial awareness through stereoscopic vision but also improves image quality, leading to more precise and efficient task performance (Prendergast et al., 2009; Saldana et al., 2020).

The growing interest in VR headsets is evident in the exponential rise in medical research publications on the subject, with 474 articles published between 1984 and 2018, compared to 1394 between 2018 and 2024. Whilst these technologies have been widely adopted in medicine—particularly in surgical and cardiology training for pre‐procedural planning (Lareyre et al., 2021; Silva et al., 2018; Tursø‐Finnich et al., 2023)—their application in dentistry remains relatively underexplored. However, recent studies suggest promising potential (Collaço et al., 2021; Rodrigues et al., 2022; Saldana et al., 2020). For instance, Rodrigues et al. investigated a series of fully immersive haptic exercises focused on Class I and II cavity preparation (Rodrigues et al., 2022, 2023). Their study found that students trained with VR headsets exhibited a significant reduction in milling time by the third session, suggesting that immersive haptic simulation could be a valuable tool in operative dentistry education (Rodrigues et al., 2022, 2023). Similarly, Collaço et al. demonstrated that students trained with VR headsets in a haptic simulator performed IAB anaesthesia more quickly and accurately than those trained in non‐immersive conditions (Collaço et al., 2021). More recently, Samuel et al. assessed the benefits of the VARIANT haptic simulator for IAB anaesthesia training. Their findings indicated that students trained with both a VR headset and haptic simulation were not only more confident but also more successful in their first patient‐administered anaesthesia injections compared with those trained using traditional methods (Samuel et al., 2024). These findings suggest that integrating immersive VR and haptic technologies into preclinical dental education may significantly enhance students' motor skill acquisition, particularly for complex procedures like IAB anaesthesia (Samuel et al., 2024). However, immersive haptic simulation is still in its early stages, and further research is needed to explore its potential for other procedures, such as prosthetic preparations and access cavity formation.

To address this gap, we conducted a comparative crossover study to: (1) evaluate the impact of a full immersion in haptic simulation on access cavity preparation, focusing on student performance measured through haptic parameters; (2) assess students' perceptions of immersive VR‐based training; and (3) analyse potential side effects associated with VR headset use. We hypothesize that students trained in an immersive environment will demonstrate superior outcomes in terms of progression, accuracy, speed of execution, and overall quality of access cavity preparation compared with those trained in non‐immersive settings.

## MATERIALS AND METHODS

### Ethical approval and participants

This comparative crossover study was approved by the Research Ethics Committee of Nantes University (IRB No.: IORG0011023). Prior to participation, all students were fully informed about the study protocol and provided written consent. The study included 90 third‐year dental students from the Dental Faculty of Nantes University, comprising 66 women and 24 men, with a mean age of 20.80 ± 0.74 years. To ensure participant safety and minimize confounding factors, students with visual impairments, severe migraines, severe anxiety, motor disorders, or a history of motion sickness were excluded from the study.

Of the participants, seven had prior experience with VR headsets (Oculus Quest, Meta Platforms Technologies) in various contexts, including: entertainment (e.g., video games, wind tunnel flight simulators, amusement parks, drone piloting), cultural experiences (e.g., museums, exhibitions), medical applications (e.g., physiotherapy), or training programmes (e.g., safety and emergency response simulations).

### Pre‐training

Before the experiment, participants attended four 20‐min hands‐on training sessions to familiarize themselves with the VirTeaSy Dental® haptic simulator (VirTeaSy, HRV Simulation). This training period was deemed sufficient for participants to gain autonomy in using the simulator across a series of scheduled exercises. During each session, participants practiced as many times as needed, tracking their progress throughout. The training focused on simulator handling, manual dexterity using the force feedback arm, fundamental operative dentistry skills, and access cavity preparation on a virtual mandibular molar. Participants received detailed instructions on performing the assessment exercises, using the VR headset, achieving the required objectives, and understanding the performance evaluation criteria measured through the simulator's haptic parameters. The VR headset (Occulus Quest, Meta Platforms Technologies) used in this study features six degrees of positional freedom, allowing users to observe the virtual environment, orient their heads, and move in corresponding directions for a fully immersive experience (Kim et al., 2014; Sukotjo et al., 2021).

### Experimental protocol

**FIGURE 1:**
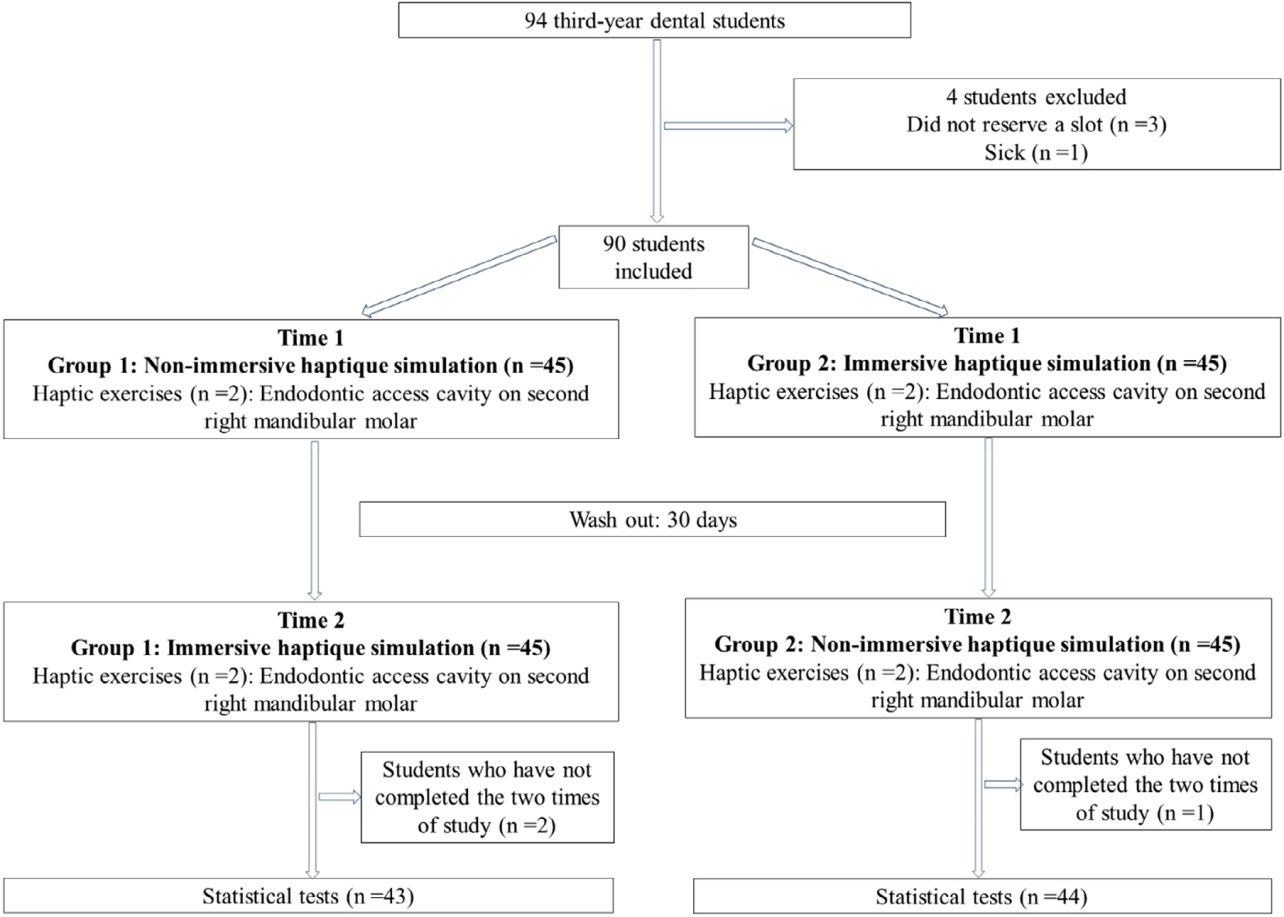
Flowchart of the study design.

The study followed a crossover design, as illustrated in Figure 1, with each participant serving as their own control. The 90 students were randomly assigned to two parallel groups (G1 and G2) in a 1:1 ratio. Amongst those with prior experience using a VR headset, three were assigned to Group 1 and four to Group 2 after randomization. Beyond the initial training sessions, each participant completed two haptic exercises focused on access cavity preparation on a right mandibular second molar. Each evaluation session lasted 20 minutes, consisting of two consecutive 10‐minute exercises. An exercise was considered unsuccessful if the participant achieved less than 90% overall progress or less than 85% accuracy within the allotted time (Bandiaky, Loison, et al., 2024; Urbankova et al., 2013, 2022; Urbankova & Engebretson, 2011). Consequently, students could accumulate between zero and two failures per session. Successful completion required precise adherence to predefined contours, the creation of an access opening into the pulp chamber, removal of the pulp chamber roof, and careful avoidance of any damage to the pulp chamber floor.

### Phase 1 (time 1: Non‐immersive for G1, immersive VR for G2)

One week following the training session, at Time 1 (T1), participants in Group 1 (G1, *n* = 45) completed two access cavity exercises on the VirTeaSy Dental® simulator without the use of a VR headset, whilst participants in Group 2 (G2, *n* = 45) performed the same exercises using the VR headset. All participants successfully completed their assessments within a 6‐week period.

### Phase 2 (time 2: Immersive VR for G1, non‐immersive for G2)

To minimize potential learning curve bias, a 30‐day washout period was implemented. Previous studies indicate that significant improvements in motor skills typically occur after three consecutive exercises (Urbankova et al., 2013; Vincent et al., 2022). In the second phase (T2), the groups switched conditions: G1 performed the exercises with the VR headset, whilst G2 completed them without it. The same protocol and exercises from T1 were replicated. As in the first phase, all participants completed their assessments within a 6‐week period.

It is important to note that although the evaluators were not blinded, the access cavity exercises were conducted within a closed session, accessible only via a code and an identifier. To reduce performance bias, no communication regarding performance parameters occurred between the student and the evaluator during the assessments.

### Haptic parameters measured

The participants' performance was assessed based on several key parameters, recorded by the VirTeaSy Dental® simulator. These included: (i) total time, which was the cumulative duration the bur remained in continuous rotation, regardless of whether the participant was actively engaged in the task; (ii) milling time, which referred to the actual time spent removing tissue; (iii) overall progress, measured as the percentage of target tissue successfully removed during the exercise; (iv) accuracy, defined as the participant's ability to stay focused on the target area and remove only the intended tissue; (v) internal volume (IV), which quantified the tissue removed within the designated target area; and (vi) outside volume (OV), which indicated the unintended (iatrogenic) tissue removed outside the target area. Data for these haptic parameters were collected and recorded by a student who was not part of the research team to ensure unbiased data collection.

### Participants' feedback and experiences

Participants' perceptions of the VirTeaSy Dental®, both with and without the VR headset, were gathered through four self‐administered questionnaires (Tables S1–S4). These questionnaires were based on the ICTE (Information and Communication Technologies for Teaching) acceptance scale (Baron et al., 2007). The ICTE framework covers a broad spectrum of technologies and educational practices used in various pedagogical settings, both in‐person and remotely (Baron et al., 2007). The ICTE scale provides a set of general criteria to analyse students' perceptions and their adoption of technology, offering valuable insights into factors that could either promote or hinder the tool's acceptance (Caron & Heutte, 2017). Although the questionnaire had not been specifically validated for dental education, its adaptation was carried out by a team of social science experts specializing in the study of human interaction with technology in educational contexts. After each session (with and without the VR headset), a questionnaire was administered to assess ease of use, participant preferences, and any side effects experienced. Additionally, semi‐structured interviews were conducted to further explore participants' experiences.

### Statistical analysis

The statistical analyses were conducted by an external member of the project to ensure impartiality in the results. All analyses were performed using GraphPad® Prism software (GraphPad Software). Descriptive statistics for qualitative variables were reported as percentages and counts, whilst quantitative variables were summarized using means, standard deviations, medians, and the range of minimum and maximum values. To compare intra‐ and inter‐group results, a T‐test was employed, provided normality of the data distribution was confirmed. In cases where the data did not meet the normality assumption (as assessed using the Shapiro–Wilk test), non‐parametric tests were applied: the Wilcoxon test for paired samples and the Mann–Whitney test for independent groups. This approach was also used in additional analyses examining participants' performance based on their prior experience with VR headsets (Wilcoxon test). Questionnaire responses were analysed through flat sorting, categorizing results based on ease of use, participant preferences, and any reported side effects.

## RESULTS

### Intra‐group comparison of haptic parameters

The intra‐group comparison of haptic parameters between T1 and T2 is summarized in Tables 1 and 2. As given in Table 1, students in G1 performed better in the non‐immersive environment compared with the immersive VR condition. Significant differences were observed in several key parameters, including total time (*p* < .05), milling time (*p* < .05), overall progress (*p* < .05), and internal volume removed (*p* < .05). Similarly, Table 2 presents that the students in G2 also achieved superior performance in the non‐immersive environment. However, no statistically significant differences (*p* > .05) were observed between the immersive and non‐immersive conditions for the majority of the haptic parameters. The exceptions were total time, overall progress, and inside volume removed, where significant differences (*p* < .05) were found. The Wilcoxon test indicated significant differences (*p* < .05) in the medians and quartiles for these three parameters. Overall, regardless of the phase (T1 or T2) or group (G1 or G2), the results consistently showed that participants performed better in the non‐immersive environment. Without the VR headset, participants were able to achieve more progress in the target area, make fewer errors, and complete the endodontic access cavity exercises faster compared to their performance with the VR headset, as indicated in Tables 1 and 2. In addition, a higher number of students failed to complete the access cavity exercises within the allotted 10‐min time frame when using the VR headset, as given in Tables 1 and 2. An additional analysis was conducted to assess the impact of prior VR experience on student performance. The findings revealed that despite prior experience with VR headsets, students performed worse when using the VR headset in the haptic simulation (Table S5). Out of the 45 participants in each group, two students from G1 and one from G2 were unable to attend the final evaluation.

**TABLE 1:** Comparison of haptic parameter results for group G1 between non‐immersive (Time 1) and immersive (Time 2) conditions.

Haptic simulator parameters	G1T1_ non‐immersive (*N* = 43)			G1T2_immersive (*N* = 43)			*p*‐value
	Mean ± SD	Min‐max	Median [Q1;Q3]	Mean ± SD	Min‐max	Median [Q1;Q3]	
Total time (min)	7.54 ± 2.32*	[2.00;11.52]	8.58 [6.07;10.12]	9.50 ± 1.33*	[5.03;12.40]	10.20 [9.33;10.33]	<.05
Drilling time (min)	4.54 ± 1.40*	[1.27;8.09]	4.50 [3.42;6.10]	5.52 ± 1.46*	[2.25;9.33]	5.57 [4.28;7.18]	<.05
Target progression (%)	88.2 ± 6.3*	[64.4;93.8]	90.5 [86.9;91.7]	82.8 ± 11.3*	[49.3;99.6]	85.7 [77.7;90.6]	<.05
Accuracy (%)	80.6 ± 8.3^a^	[52.5;96.5]	82.5 [75.8;86.7]	78.0 ± 10.5^a^	[54.3;96.1]	80.2 [72.2;84.8]	>.05
Inside volume (IV, mm^3^)	175.9 ± 12.67*	[128.3;187.0]	180.3 [173.3;182.8]	165.0 ± 22.6*	[98.4;198.6]	170.9 [154.9;180.7]	<.05
Outside volume (OV, mm^3^)	45.59 ± 27.41^a^	[5.4;150.8]	36.1 [28.2;57.6]	53.01 ± 37.52^a^	[4.0;151.8]	40.3 [27.7;69.5]	>.05
Successful completion of haptic simulator exercise if overall progress reaches 90% in 10 min.	Success (n/N)		31/43 (72%)	Success (n/N)		16/43 (37%)	
	Failed (n/N)		12/43 (27%)	Failed (n/N)		27/43 (62%)	

*Note*: When comparing non‐immersive and immersive environments, several significant differences were observed. Total time and drilling time were notably longer in the immersive environment, indicating that participants required more time in this setting. Conversely, overall progression was better in the non‐immersive environment, reflecting more efficient performance. Although accuracy slightly decreased in the immersive environment, this difference was not statistically significant. A significant reduction in internal volume was observed in the immersive environment, suggesting less efficient volume removal. External volume increased slightly in the immersive environment, but this difference was not significant. Finally, the success rate was significantly lower in the immersive environment, with fewer students achieving success. Overall, students performed better in the non‐immersive haptic simulation, with statistically significant differences in most parameters, except for accuracy and external volume removal.

Abbreviations: G1, group 1; Max, maximum; Min, minimum; SD, standard deviation.

^a^ No significant difference (*p* > .05).

* Significant difference (*p* < .05).

**TABLE 2:** Comparison of haptic parameter results for group G2 between immersive (T1) and non‐immersive (T2) conditions.

Haptic simulator parameters	G2T1_immersive (*N* = 44)			G2T2_non‐immersive (*N* = 44)			p‐value
	Mean ± SD	Min‐max	Median [Q1;Q3]	Mean ± SD	Min‐max	Median [Q1;Q3]	
Total time (mn)	9.42 ± 1.36	[4.46;13.14]	10.14 [8.51;10.31]*	9.04 ± 1.38	[5.08;13.19]	9.20 [7.55;10.18]*	<.05
Drilling time (mn)	5.57 ± 1:28^a^	[2.53;9.19]	5.49 [4.46;7.08]	5.31 ± 1.30^a^	[2.49;8.43]	5.24 [4.24;6.47]	>.05
Target progression (%)	84.6 ± 8.5*	[58.1;95.0]	88.5 [79.9;90.7]*	87.5 ± 8.7^a^	[57.2;96.0]	90.1 [87.6;92.1]*	<.05
Accuracy (%)	78.9 ± 8.7^a^	[58.5;95.9]	79.8 [72.2;85.4]	81.5 ± 7.6^a^	[60.5;95.1]	82.1 [76.4;87.5]	>.05
Inside volume (IV, mm3)	168.7 ± 16.9	[115.8;189.5]	176.5 [159.4;180.8]*	174.5 ± 17.3*	[114.1;191.5]	179.7 [174.6;183.5]*	<.05
Outside volume (OV, mm3)	48.8 ± 27.1	[4.9;116.4]	43.6 [28.5;64.9]^a^	42.0 ± 22.4^a^	[8.4;109.5]	38.8 [25.5;55.4]^a^	>.05
Successful completion of haptic simulator exercise if overall progress reaches 90% in 10 min.	Success (n/N)		21/44 (47%)		Success (n/N)	31/44 (70%)	
	Failed (n/N)		23/44 (52%)		Failed (n/N)	13/44 (29%)	

*Note*: A comparative analysis of the immersive (T1) and non‐immersive (T2) environments was conducted using statistical tests for haptic parameters. For normally distributed data, such as drilling time, accuracy, and external volume, the Student's *t*‐test revealed no significant difference between T1 and T2 (*p* > .05). In contrast, for non‐normally distributed data, including total time, target progression, and internal and external volumes, the non‐parametric Wilcoxon test showed significant differences in the medians and quartiles based on the environment, with the exception of external volume (*p* < .05). Additionally, the success rate was significantly higher in the non‐immersive environment (T2) compared with the immersive environment (T1).

Abbreviations: G2, group 2; Max, maximum; Min, minimum; T1, time 1; T2, time 2.

^a^ No significant difference (*p* > .05).

* Significant difference between medians.

### Intergroup comparison of haptic parameters

The comparison of haptic parameters between G1 and G2 during both study periods (T1 and T2) revealed performance differences under immersive and non‐immersive conditions (Table S1). At T1, G1 demonstrated better overall performance in the non‐immersive environment compared to G2 in the immersive VR condition. Specifically, G1 outperformed G2 in terms of total time, overall progress, and internal volume removed, with statistically significant differences observed (Mann–Whitney test, *p* < .05). Conversely, at T2, G2 performed better in the non‐immersive condition compared to G1 in the immersive VR condition. Significant differences were again noted in total time, milling time, overall progress, and internal volume removed (*p* < .05). Regarding success rates, at T1, G1 showed a significantly higher success rate (72%, 31/43) than G2 (47%, 21/44). At T2, G2 had a higher success rate (70%, 31/44) compared to G1 (37%, 16/43).

### Comparison of haptic parameters between immersive (*N* = 87) and non‐immersive (*N* = 87) groups

Table 3 presents a comparison of haptic parameter results between immersive and non‐immersive haptic simulations. A statistically significant difference was found in favour of the non‐immersive simulation for most parameters (*p* < .05), with the exception of external volume removed, where no significant difference was observed (*p* > .05). Overall, the non‐immersive haptic simulation demonstrated superior performance across all haptic parameters during access cavity preparation.

**TABLE 3:** Comparison of haptic parameter outcomes between immersive and non‐immersive haptic simulations across groups.

Haptic simulator parameters	Immersive (*N* = 87)			Non‐immersive (*N* = 87)			*p*‐value
	Mean ± SD	Min‐max	Median [Q1;Q3]	Mean ± SD	Min‐max	Median [Q1;Q3]	
Total time (mn)	9.46 ± 1.34*	[4.46;13.14]	10.17 [9.01;10.32]	8.29 ± 2.11*	[2.00;13.19]	9.10 [6.52;10.14]	<.05
Drilling time (mn)	5:54 ± 1:37*	[2:25;9:33]	5:56 [4:38;7:17]	5:13 ± 1:36*	[1:27;8:43]	5:10 [3:55;6:38]	<.05
Target progression (%)	83.7 ± 10.0*	[49.3;99.6]	87.4 [78.9;90.6]	87.9 ± 7.6*	[57.2;96.0]	90.4 [87.0;91.8]	<.05
Accuracy (%)	78.4 ± 9.6*	[54.3;96.1]	80.0 [72.2;85.2]	81.0 ± 7.9	[52.5;96.5]	82.4 [75.9;86.8]	<.05
Inside volume (IV, mm^3^)	166.9 ± 19.9*	[98.4;198.6]	174.3 [157.4;180.7]	175.2 ± 15.1*	[114.1;191.5]	180.1 [173.5;183.1	<.05
Outside volume (OV, mm^3^)	50.9 ± 32.6^a^	[4.0;151.8]	40.7 [28.2;69.5]	43.8 ± 24.9^a^	[5.4;150.8]	37.3 [26.8;57.0]	>.05

*Note*: In comparing immersive and non‐immersive environments, significant differences were found across several parameters for endodontic access cavity preparation. Total time was significantly longer in the immersive group compared with the non‐immersive group (*p* < .05). Drilling time also increased in the immersive group (*p* < .05). Target progression was slightly but significantly lower in the immersive group (*p* < .05). Accuracy showed only a minor difference, which was not statistically significant (*p* < .05). Internal volume removed was lower in the immersive group (*p* < .05), whilst no significant difference was observed for external volume removed. These results suggest that non‐immersive haptic simulation is superior in most parameters for endodontic access cavity preparation, except for external volume removal.

Abbreviations: Max, maximum; Min, minimum; SD, standard deviation.

^a^ No significant difference (*p* > .05).

* Significant difference.

### Overall ease of use of VirTeaSy dental®

A total of 174 responses were analysed to evaluate students' experiences using the VirTeaSy Dental® simulator, both with and without the VR headset. When the VR headset was introduced, students reported a decrease in the perceived ease of use, whilst switching back to the headless mode led to an improvement (Figure S1). Additionally, students found the working position to be less restrictive without the VR headset (Figure S2). Regarding the haptic arm, G1 experienced increased difficulty during T2, whilst G2 reported an improvement in ease of use when the simulator was operated without the VR headset. In general, the inclusion of the VR headset negatively impacted both the ease of use and the handling of the haptic arm.

### VirTeaSy dental® user preferences

This section explores students' perceptions of the realism of sensations provided by the VirTeaSy Dental® simulator and their preferences for using the system with or without the VR headset. At the conclusion of the first phase, most students in both G1 and G2 expressed enjoyment of their experience with the simulator. The introduction of the VR headset had a noticeable impact on the perceived realism of the simulation (Figure S3) and the physical sensations, such as contact with teeth, enamel, dentin, and milling with various tools (Figure S4). Overall, most students preferred using the simulator without the VR headset, reporting better outcomes when the system was used in non‐immersive mode.

### Side effects associated with use of the VR headset

Approximately, 20% of students in G1 and 18% in G2 reported experiencing side effects during the use of the VR headset. These included dizziness, nausea, headaches, numbness, and muscle fatigue in the neck area.

## DISCUSSION

This comparative crossover study aimed to assess the impact of full immersion in haptic simulation for endodontic access cavity preparation, focusing on student performance, perceptions, and side effects associated with VR headset use. Our findings showed that students performed better on average when using the VirTeaSy Dental® simulator without a VR headset. Specifically, students exhibited greater accuracy, progressed more quickly toward the target, and removed less iatrogenic tissue in the non‐immersive environment. These results suggest that a non‐immersive setup provides clear advantages in facilitating the acquisition of motor skills, particularly in complex procedures like access cavity preparation. In contrast to our initial hypothesis, which proposed that students trained in immersive VR conditions would perform better, the results led us to reject this assumption. Instead, the combination of VR headsets and haptic technology impacted negatively performance in complex exercises. Interestingly, despite prior VR experience amongst some students (G1, *n* = 3; G2, *n* = 4), their performance when using the VR headset for access cavity preparation was still suboptimal. This suggests that VR headsets may introduce additional challenges in mastering specific dental procedures, particularly when complex haptic feedback is involved. Our study's findings highlight the difficulties students face in immersive haptic simulations. Visual discomfort, cognitive overload, and blurred vision are common complaints when using VR headsets, as discussed by Yuan et al. (2018). These issues impede students' ability to clearly visualize critical areas for milling, which is essential for accurate cavity preparation.

Furthermore, students reported struggling with poor depth perception and difficulty controlling the rotating virtual instruments when using VR headsets, which are crucial for performing precise tasks in endodontic procedures. Exacerbating these issues were high light intensity and spray effects, which caused visual opacity and further impaired students' ability to navigate the virtual environment. The density of the hard tissues also posed a challenge, as their lower resistance led to excessive tissue removal, even with small errors. These compounded difficulties resulted in slower task completion, more frequent errors, and an increased number of failures when students used the VR headset for the exercises. Other factors contributing to the decline in student performance when using the VR headset are discussed in the article by Loison (2024).

It is essential to note that the observed performance improvement cannot be solely attributed to familiarization with the simulator. The crossover design of the experiment ensures that each participant serves as their own control and experiences the same familiarization period. If familiarization were the primary factor driving performance improvement, we would expect similar results across all conditions. However, participants who used the VR headset in Phase 2, following the washout period, showed a decline in performance compared to Phase 1, which started 1 week earlier. To the best of our knowledge, the only study comparing immersive versus non‐immersive haptic simulation for motor skills acquisition in IAB anaesthesia was conducted by Collaço et al. (Collaço et al., 2021). This study assessed technical skills related to needle insertion, perceptions of syringe manipulation, and side effects associated with VR headset use. The authors concluded that students in immersive groups with activated haptic feedback performed the anaesthetic procedure more quickly, accurately, and confidently than those in non‐immersive groups on the VIDA Odonto® simulator (São Paulo, Brazil). However, our findings, which indicate negative effects of VR headset use in haptic simulation for a completely different procedure, contradict these results. Collaço et al. focused on an IAB anaesthesia scenario that requires precision in needle insertion within a specific target area, with the aid of intra‐oral landmarks, and has a relatively larger work volume (Collaço et al., 2021). This simpler scenario differs significantly from ours, which involves milling a smaller work area that demands precise visualization of the occlusal face and tooth walls, along with accurate handling of rotating virtual instruments. Our scenario, which demands intricate, precise movements and detailed visualization, proves considerably more challenging to execute in full immersion due to the challenges associated with wearing the VR headset. Based on our findings, immersive haptic simulation may be better suited for simpler tasks that involve working on a single axis. For more complex procedures like endodontic access cavity preparation, the technology needs substantial refinement before it can be reliably integrated into training.

In contrast, a study exploring the benefits of immersive haptic simulation in operative dentistry reported promising results, including reduced total and milling time after three training sessions on Class I and II cavities (Rodrigues et al., 2023). However, the study did not compare these parameters under non‐immersive conditions, preventing a direct comparison with our findings. Similarly, a recent study by Samuel et al. on the VARIANT simulator for IAB anaesthesia in immersive conditions found that students using the simulator felt more confident, required fewer syringe readjustments, and had greater success in anaesthetising patients compared to those using traditional training methods (Samuel et al., 2024). Whilst this study highlights the potential of immersive virtual reality in preclinical dental training, particularly for IAB anaesthesia, it did not include a comparison with non‐immersive environments. The existing literature suggests potential benefits of immersive haptic simulation, but without direct comparisons to non‐immersive conditions, it may lead to premature adoption of the technology, overlooking its limitations, as shown in our study.

Our findings indicate that students preferred the non‐immersive environment when using the VirTeaSy Dental®, with enjoyment decreasing in the second phase. Several factors can contribute to this decline, as reported in this article by Loison (Loison, 2024), which provides a qualitative analysis of the data. These include discomfort with the VR headset (headaches, dizziness, blurred vision, and a loss of control), fatigue, frustration with the virtual environment, difficulty adjusting to the simulator, and challenges with depth perception. Some students also found the transition to the VR headset disruptive and felt it reduced realism. This contrasts with the findings of Collaço et al. (Collaço et al., 2021), who reported a preference for immersive haptic simulation. However, there are no other studies comparing user preferences between immersive and non‐immersive haptic simulation in dental training, specifically in terms of user preference.

Some adverse psychological effects, such as dizziness, nausea, or migraines, were reported by a small number of students. These effects have been documented in other studies as well (Dużmańska et al., 2018). For instance, in their systematic review of 12 studies, Tursø‐Finnich et al. identified symptoms, such as dizziness, nausea, headaches, discomfort, and visual disturbances (Tursø‐Finnich et al., 2023). Whilst these effects were minimal in our study, they should not be overlooked by VR headset developers, as they could pose a barrier to the broader adoption of immersive haptic simulation in preclinical dental training (Kennedy et al., 2010).

Our study has several limitations that must be considered. First, the VR headset's resolution limits the minimum distance at which users can clearly perceive objects in the virtual environment, affecting precision during complex tasks like access cavity preparation. Whilst solutions such as auto‐zoom could improve visual perception, they may undermine the sense of immersion. Additionally, the monocentric nature of our study restricts the generalizability of the findings to other student populations with varying levels of experience or from different institutions. Moreover, our focus on access cavity preparation may limit the applicability of the results to other clinical scenarios. Another limitation is the absence of continuous performance measurements, which would allow for a more detailed analysis of participants' progress under both immersive and non‐immersive conditions. Finally, participants' prior experience with VR may introduce selection bias, potentially influencing the results. To address these limitations, future research should include a more diverse sample, explore different clinical scenarios, and incorporate continuous performance tracking to better assess participants' learning trajectories.

## CONCLUSION

This study reveals that students perform better on all haptic parameters when using the VirTeaSy Dental® without a VR headset during endodontic access cavity exercises. Although immersive haptic simulation shows potential, its effectiveness for complex tasks remains limited and may even hinder performance in a training context. The side effects reported by students, along with the higher failure rates observed, underscore the need for significant improvements in the integration of immersive virtual reality with haptic technology before its widespread adoption in preclinical dental education.

## AUTHOR CONTRIBUTIONS

Octave N. Bandiaky: Conceptualization; data curation; investigation; methodology; writing – original draft; writing – review and editing. Valériane Loison: Study design; participant recruitment; training; experimentation; evaluation; manuscript drafting. Christelle Volteau: Statistical analyses; review and editing; manuscript drafting. Raphaëlle Crétin‐Pirolli: Supervision; validation; visualization; writing – review and editing. Sébastien George: Funding acquisition; project administration; resources; supervision; validation; study design; training; experimentation; evaluation. Assem Soueidan: Methodology; supervision; validation; visualization; manuscript drafting. Laurent Le Guehennec: Investigation; methodology; supervision; validation; visualization; writing – original draft; writing – review and editing; manuscript drafting.

## CONFLICT OF INTEREST STATEMENT

None; the authors declare no conflict of interest.

## ETHICS STATEMENT

This study was approved by the Ethics Committee of Nantes Université. All participants gave their informed consent before participating. The study was conducted in accordance with the principles of the Declaration of Helsinki.

## Supporting information


Figure S1.

Table S1.

## Data Availability

All raw data will be available upon request from the corresponding author.
