# Supplementary material for: Benefits of using immersive virtual reality in haptic dental simulation for endodontic access cavity training: A comparative crossover study
Source: Int Endod J. 2025 May 12;59(6):1110–21. doi: 10.1111/iej.14252 (PMC13158534; doi:10.1111/iej.14252)
Supplement: Supplementary file 1 — Figure S1. [file IEJ-59-1110-s001.docx]

**Figure S1**


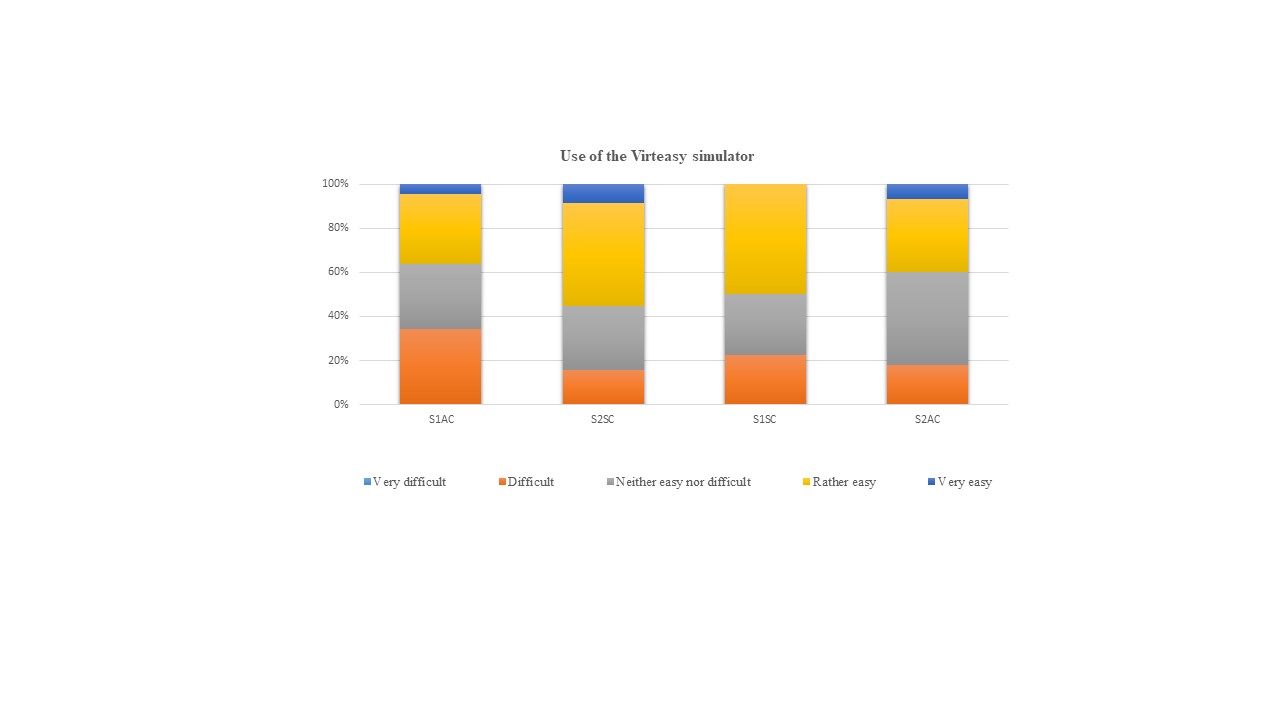


**Figure S2**


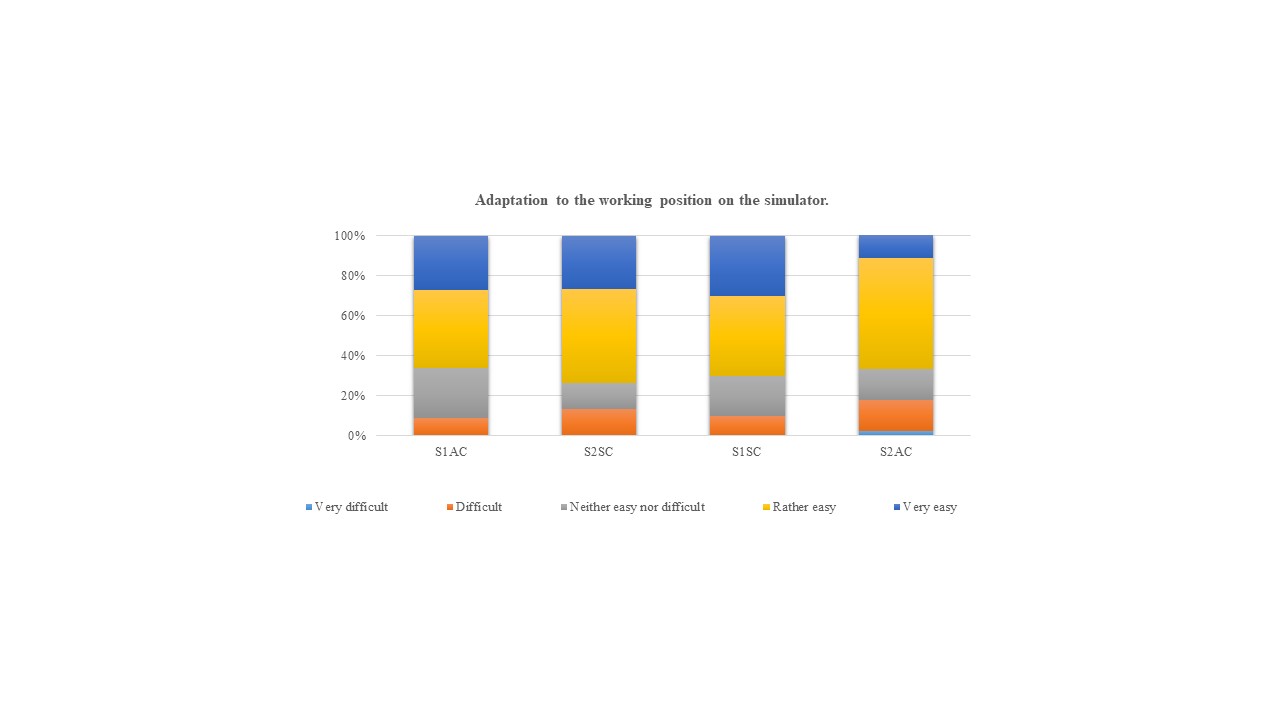


**Figure S3**


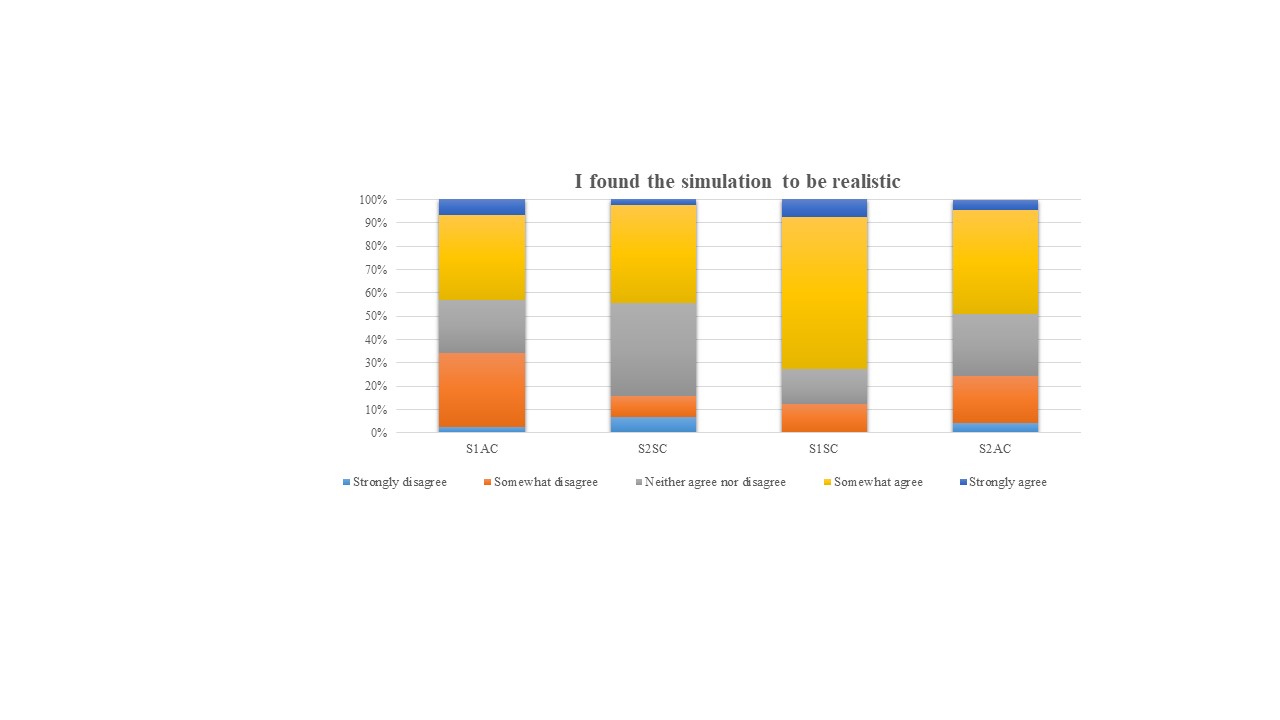


**Figure S4**


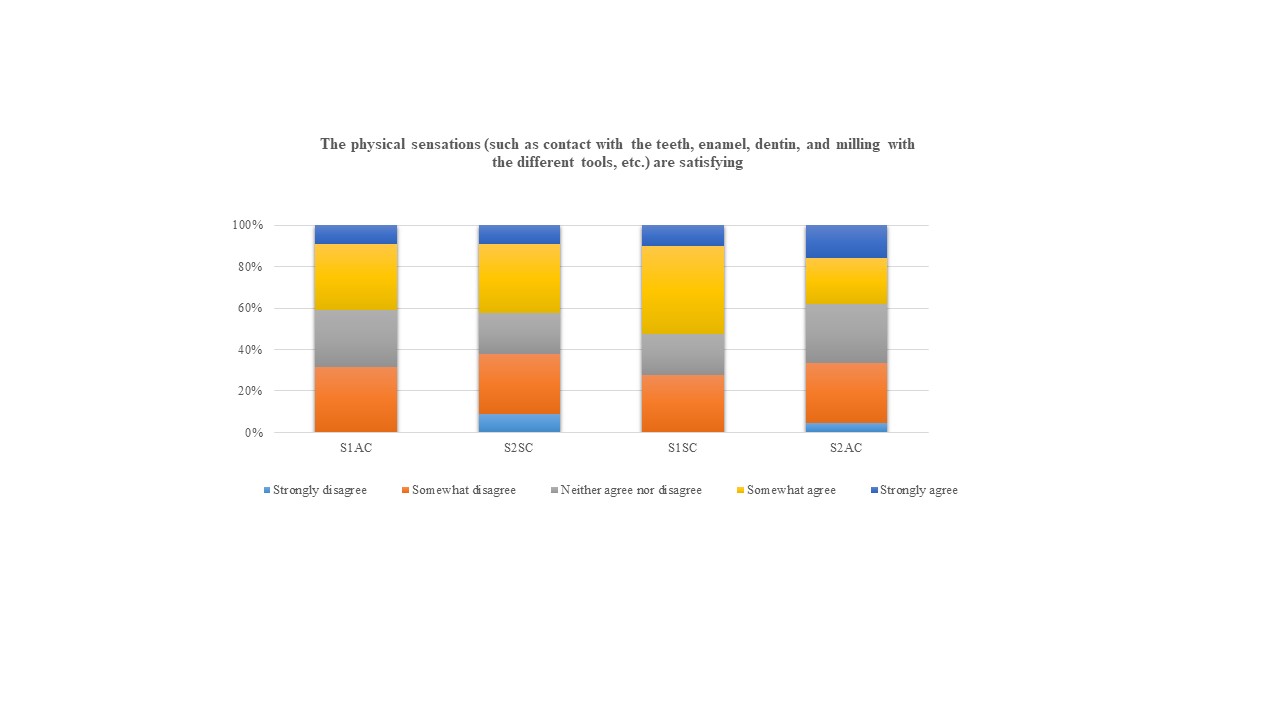


**Legend for supplementary figures**

**Figure S1.** Ease of use of the VirTeaSy Dental^®^ simulator.

**Figure S2.** Ease of adapting to the working position on the simulator.

**Figure S3.** Appreciation of the simulation's realism.

**Figure S4.** Satisfaction with physical sensations.
