# Supplementary material for: Benefits of using immersive virtual reality in haptic dental simulation for endodontic access cavity training: A comparative crossover study
Source: Int Endod J. 2025 May 12;59(6):1110–21. doi: 10.1111/iej.14252 (PMC13158534; doi:10.1111/iej.14252)
Supplement: Supplementary file 2 — Table S1. [file IEJ-59-1110-s002.docx]

Table S1. CREN - Questionnaire on the use of the VirTeasy Simulator S1 without VR (T1-G1-without VR)

You have used a haptic simulator in your practical sessions.

The goal of this questionnaire is to gather your feedback to improve your training through the integration of new digital tools. You have just completed your first practical evaluation session with the VirTeasy simulator, without the virtual reality (VR) headset. This questionnaire is anonymous and will take no more than 5 minutes to complete. There are 12 questions in this questionnaire.

| General Information | |
| --- | --- |
| What is your VirTeasy ID? | Please write your response here: |
| What is your gender? | Please select one response below:  Female  Male  Non-gendered |
| What is your age? | Please write your response here: |
| Have you already used the VirTeasy simulator in the first semester? | Yes  No |
| Have you ever used a VR headset outside of the VirTeasy simulator? | Yes  No |
| If yes, in what activities have you used a VR headset? | Please write your response here: |

| General Questions on the Use of the VirTeasy Simulator | | | | | |
| --- | --- | --- | --- | --- | --- |
| What are your general impressions on the following points? | | | | | |
| Select the appropriate response for each item | Strongly Disagree | Somewhat Disagree | Neutral | Somewhat Agree | Strongly Agree |
| I enjoyed using the VirTeasy simulator. |  |  |  |  |  |
| I would like to use the VirTeasy simulator again during my training. |  |  |  |  |  |
| My posture while using the tool is satisfactory. |  |  |  |  |  |
| The exercises on the simulator were useful for learning the milling technique. |  |  |  |  |  |
| The physical sensations (contact with teeth, enamel, dentine, milling with different tools, etc.) are satisfactory. |  |  |  |  |  |
| Viewing the tooth in three cross-sectional planes was useful to me. |  |  |  |  |  |
| The milling time indicator was useful to me. |  |  |  |  |  |
| The target progress indicator was useful to me. |  |  |  |  |  |
| The precision indicator was useful to me. |  |  |  |  |  |
| I found the simulation realistic. |  |  |  |  |  |

| Your Experience with the VirTeasy Simulator on the Following Aspects: | | | | | |
| --- | --- | --- | --- | --- | --- |
| Select the appropriate response for each item | Very difficult | Difficult | Neutral | Easy | Very easy |
| Using the VirTeasy simulator |  |  |  |  |  |
| Handling the haptic arm |  |  |  |  |  |
| Adapting to the working position on the simulator |  |  |  |  |  |
| Using the 3D mouse |  |  |  |  |  |
| Performing the exercise: First attempt |  |  |  |  |  |
| Performing the exercise: Second attempt |  |  |  |  |  |

| Do you have any comments on the use of the VirTeasy simulator? Please write your response here: |
| --- |
| How would you like to use the VirTeasy simulator in the future? Please write your response here: |
| In the future, what features would you like to improve or add to the VirTeasy simulator? Please write your response here: |

| Additional Questions on the Use of the VirTeasy Simulator | | | | | |
| --- | --- | --- | --- | --- | --- |
| What do you think about the following statements regarding the VirTeasy simulator? | | | | | |
| Select the appropriate response for each item | Strongly Disagree | Somewhat Disagree | Neutral | Somewhat Agree | Strongly Agree |
| The simulator improves my ability to apply what I have learned during my training. |  |  |  |  |  |
| With the simulator, I think it is easier to train. |  |  |  |  |  |
| The simulator allows me to learn faster. |  |  |  |  |  |
| I use the simulator because it is required. |  |  |  |  |  |
| It is easy to learn how to use the simulator. |  |  |  |  |  |
| With the simulator, learning is easier. |  |  |  |  |  |
| I find the simulator easy to use. |  |  |  |  |  |
| I use the simulator because other trainees use it. |  |  |  |  |  |
| Using the simulator makes me more efficient. |  |  |  |  |  |
| The simulator is useful for my learning. |  |  |  |  |  |

Thank you for participating in this survey!

Submit your questionnaire. Thank you for completing this questionnaire.

Table S2. CREN - Questionnaire on the use of the VirTeasy Simulator S1 with VR (T1-G2-VR)

You have used a haptic simulator in your practical sessions.

The goal of this questionnaire is to gather your feedback to improve your training through the integration of new digital tools. You have just completed your first practical evaluation session with the VirTeasy simulator, using the virtual reality (VR) headset. This questionnaire is anonymous and will take no more than 5 minutes to complete. There are 13 questions in this questionnaire.

| General Information | |
| --- | --- |
| What is your VirTeasy ID? | Please write your response here: |
| What is your gender? | Please select one response below:  Female  Male  Non-gendered |
| What is your age? | Please write your response here: |
| Have you already used the VirTeasy simulator in the first semester? | Yes  No |
| Have you ever used a VR headset outside of the VirTeasy simulator? | Yes  No |
| If yes, in what activities have you used a VR headset? | Please write your response here: |

| General Questions on the Use of the VirTeasy Simulator | | | | | |
| --- | --- | --- | --- | --- | --- |
| What are your general impressions on the following points? | | | | | |
| Select the appropriate response for each item | Strongly Disagree | Somewhat Disagree | Neutral | Somewhat Agree | Strongly Agree |
| I enjoyed using the VirTeasy simulator. |  |  |  |  |  |
| I would like to use the VirTeasy simulator again during my training. |  |  |  |  |  |
| My posture while using the tool is satisfactory. |  |  |  |  |  |
| The exercises on the simulator were useful for learning the milling technique. |  |  |  |  |  |
| The physical sensations (contact with teeth, enamel, dentine, milling with different tools, etc.) are satisfactory. |  |  |  |  |  |
| Viewing the tooth in three cross-sectional planes was useful to me. |  |  |  |  |  |
| The milling time indicator was useful to me. |  |  |  |  |  |
| The target progress indicator was useful to me. |  |  |  |  |  |
| The precision indicator was useful to me. |  |  |  |  |  |
| I found the simulation realistic. |  |  |  |  |  |
| I could easily navigate the virtual environment. |  |  |  |  |  |
| I experienced side effects while using the VR headset. |  |  |  |  |  |

| If you experienced any side effects while using the VR headset, could you specify which ones? Please write your response here: |
| --- |

| Your Experience with the VirTeasy Simulator on the Following Aspects: | | | | | |
| --- | --- | --- | --- | --- | --- |
| Select the appropriate response for each item | Very difficult | Difficult | Neutral | Easy | Very easy |
| Using the VirTeasy simulator |  |  |  |  |  |
| Handling the haptic arm |  |  |  |  |  |
| Adapting to the working position on the simulator |  |  |  |  |  |
| Using the 3D mouse |  |  |  |  |  |
| Using the controller (for controlling the tablet in the virtual environment) |  |  |  |  |  |
| Navigating the virtual environment |  |  |  |  |  |
| Using the VR headset |  |  |  |  |  |
| Performing the exercise: First attempt |  |  |  |  |  |
| Performing the exercise: Second attempt |  |  |  |  |  |

| Do you have any comments on the use of the VirTeasy simulator? Please write your response here: |
| --- |
| How would you like to use the VirTeasy simulator in the future? Please write your response here: |
| In the future, what features would you like to improve or add to the VirTeasy simulator? Please write your response here: |

| Additional Questions on the Use of the VirTeasy Simulator | | | | | |
| --- | --- | --- | --- | --- | --- |
| What do you think about the following statements regarding the VirTeasy simulator? | | | | | |
| Select the appropriate response for each item | Strongly Disagree | Somewhat Disagree | Neutral | Somewhat Agree | Strongly Agree |
| The simulator improves my ability to apply what I have learned during my training. |  |  |  |  |  |
| With the simulator, I think it is easier to train. |  |  |  |  |  |
| The simulator allows me to learn faster. |  |  |  |  |  |
| I use the simulator because it is required. |  |  |  |  |  |
| It is easy to learn how to use the simulator. |  |  |  |  |  |
| With the simulator, learning is easier. |  |  |  |  |  |
| I find the simulator easy to use. |  |  |  |  |  |
| I use the simulator because other trainees use it. |  |  |  |  |  |
| Using the simulator makes me more efficient. |  |  |  |  |  |
| The simulator is useful for my learning. |  |  |  |  |  |

Thank you for participating in this survey!

Submit your questionnaire. Thank you for completing this questionnaire.

**Table S3**. CREN - Questionnaire on the Use of the VirTeasy Simulator S2 with VR headset (T2-G1-VR)

You have used a haptic simulator in your practical sessions. The purpose of this questionnaire is to gather your feedback in order to improve your training through the integration of new digital tools. You have just completed your second practical evaluation session with the VirTeasy simulator, this time with the VR headset. This questionnaire is anonymous and will take no more than 5 minutes to complete. There are 11 questions in this questionnaire.

| General Information | |
| --- | --- |
| What is your VirTeasy ID? | Please write your response here: |

| General Questions on the Use of the VirTeasy Simulator | | | | | |
| --- | --- | --- | --- | --- | --- |
| What are your general impressions on the following points? | | | | | |
| Choose the appropriate response for each item | Strongly Disagree | Somewhat Disagree | Neutral | Somewhat Agree | Strongly Agree |
| I enjoyed using the VirTeasy simulator. |  |  |  |  |  |
| I would like to use the VirTeasy simulator again during my training. |  |  |  |  |  |
| My posture while using the tool is satisfactory. |  |  |  |  |  |
| The exercises on the simulator were useful for learning the milling technique. |  |  |  |  |  |
| The physical sensations (contact with teeth, enamel, dentine, milling with different tools, etc.) are satisfactory. |  |  |  |  |  |
| Viewing the tooth in three cross-sectional planes was useful to me. |  |  |  |  |  |
| The milling time indicator was useful to me. |  |  |  |  |  |
| The target progress indicator was useful to me. |  |  |  |  |  |
| The precision indicator was useful to me. |  |  |  |  |  |
| I found the simulation realistic. |  |  |  |  |  |
| I could easily navigate the virtual environment. |  |  |  |  |  |
| I experienced side effects while using the VR headset. |  |  |  |  |  |

| If you experienced any side effects while using the VR headset, could you specify which ones? Please write your response here: |
| --- |

| Your Experience with the VirTeasy Simulator on the Following Aspects: | | | | | |
| --- | --- | --- | --- | --- | --- |
| Choose the appropriate response for each item | Very difficult | Difficult | Neutral | Easy | Very easy |
| Using the VirTeasy simulator |  |  |  |  |  |
| Handling the haptic arm |  |  |  |  |  |
| Adapting to the working position on the simulator |  |  |  |  |  |
| Using the 3D mouse |  |  |  |  |  |
| Using the controller (for controlling the tablet in the virtual environment) |  |  |  |  |  |
| Navigating the virtual environment |  |  |  |  |  |
| Using the VR headset |  |  |  |  |  |
| Performing the exercise: First attempt |  |  |  |  |  |
| Performing the exercise: Second attempt |  |  |  |  |  |

| Do you have any comments on the use of the VirTeasy simulator? Please write your response here: |
| --- |
| How would you like to use the VirTeasy simulator in the future? Please write your response here: |
| In the future, what features would you like to improve or add to the VirTeasy simulator? Please write your response here: |

| Additional Questions on the Use of the VirTeasy Simulator | | | | | |
| --- | --- | --- | --- | --- | --- |
| What do you think about the following statements regarding the VirTeasy simulator? | | | | | |
| Choose the appropriate response for each item | Strongly Disagree | Somewhat Disagree | Neutral | Somewhat Agree | Strongly Agree |
| The simulator improves my ability to apply what I have learned during my training. |  |  |  |  |  |
| With the simulator, I think it is easier to train. |  |  |  |  |  |
| The simulator allows me to learn faster. |  |  |  |  |  |
| I use the simulator because it is required. |  |  |  |  |  |
| It is easy to learn how to use the simulator. |  |  |  |  |  |
| With the simulator, learning is easier. |  |  |  |  |  |
| I find the simulator easy to use. |  |  |  |  |  |
| I use the simulator because other trainees use it. |  |  |  |  |  |
| Using the simulator makes me more efficient. |  |  |  |  |  |
| The simulator is useful for my learning. |  |  |  |  |  |

| What are your impressions on the following points? | | | | | |
| --- | --- | --- | --- | --- | --- |
| Choose the appropriate response for each item | Strongly Disagree | Somewhat Disagree | Neutral | Somewhat Agree | Strongly Agree |
| I prefer to use the VirTeasy simulator without the VR headset. |  |  |  |  |  |
| I prefer to use the VirTeasy simulator with the VR headset. |  |  |  |  |  |
| I prefer not to use the VirTeasy simulator. |  |  |  |  |  |

**Regardless of your answer, why do you feel this way?**

Please write your response here:

**Participation in Further Studies**

We are looking for students to continue our study on the use of the VirTeasy simulator. Would you be willing to discuss this with us? *

Yes, I am willing.

No, I am not interested.

Thank you for participating in this survey!

Submit your questionnaire. Thank you for completing this questionnaire.

Table S4. Questionnaire on the Use of the VirTeasy Simulator S2 without VR (T2-G2-without VR)

You have used a haptic simulator in your practical sessions. The purpose of this questionnaire is to gather your feedback in order to improve your training through the integration of new digital tools. You have just completed your second practical session with the VirTeasy simulator in Semester 2, this time without the VR headset. This questionnaire is anonymous and will take no more than 5 minutes to complete. There are 10 questions in this questionnaire.

| General Information | |
| --- | --- |
| What is your VirTeasy ID? | Please write your response here: |

| General Questions on the Use of the VirTeasy Simulator | | | | | |
| --- | --- | --- | --- | --- | --- |
| What are your general impressions on the following points? | | | | | |
| Choose the appropriate response for each item | Strongly Disagree | Somewhat Disagree | Neutral | Somewhat Agree | Strongly Agree |
| I enjoyed using the VirTeasy simulator. |  |  |  |  |  |
| I would like to use the VirTeasy simulator again during my training. |  |  |  |  |  |
| My posture while using the tool is satisfactory. |  |  |  |  |  |
| The exercises on the simulator were useful for learning the milling technique. |  |  |  |  |  |
| The physical sensations (contact with teeth, enamel, dentine, milling with different tools, etc.) are satisfactory. |  |  |  |  |  |
| Viewing the tooth in three cross-sectional planes was useful to me. |  |  |  |  |  |
| The milling time indicator was useful to me. |  |  |  |  |  |
| The target progress indicator was useful to me. |  |  |  |  |  |
| The precision indicator was useful to me. |  |  |  |  |  |
| I found the simulation realistic. |  |  |  |  |  |

| Your Experience with the VirTeasy Simulator on the Following Aspects: | | | | | |
| --- | --- | --- | --- | --- | --- |
| Choose the appropriate response for each item | Very difficult | Difficult | Neutral | Easy | Very easy |
| Using the VirTeasy simulator |  |  |  |  |  |
| Handling the haptic arm |  |  |  |  |  |
| Adapting to the working position on the simulator |  |  |  |  |  |
| Using the 3D mouse |  |  |  |  |  |
| Performing the exercise: First attempt |  |  |  |  |  |
| Performing the exercise: Second attempt |  |  |  |  |  |

| Do you have any comments on the use of the VirTeasy simulator? Please write your response here: |
| --- |
| How would you like to use the VirTeasy simulator in the future? Please write your response here: |
| In the future, what features would you like to improve or add to the VirTeasy simulator? Please write your response here: |

| Additional Questions on the Use of the VirTeasy Simulator | | | | | |
| --- | --- | --- | --- | --- | --- |
| What do you think about the following statements regarding the VirTeasy simulator? | | | | | |
| Choose the appropriate response for each item | Strongly Disagree | Somewhat Disagree | Neutral | Somewhat Agree | Strongly Agree |
| The simulator improves my ability to apply what I have learned during my training. |  |  |  |  |  |
| With the simulator, I think it is easier to train. |  |  |  |  |  |
| The simulator allows me to learn faster. |  |  |  |  |  |
| I use the simulator because it is required. |  |  |  |  |  |
| It is easy to learn how to use the simulator. |  |  |  |  |  |
| With the simulator, learning is easier. |  |  |  |  |  |
| I find the simulator easy to use. |  |  |  |  |  |
| I use the simulator because other trainees use it. |  |  |  |  |  |
| Using the simulator makes me more efficient. |  |  |  |  |  |
| The simulator is useful for my learning. |  |  |  |  |  |

| What are your impressions on the following points? | | | | | |
| --- | --- | --- | --- | --- | --- |
| Choose the appropriate response for each item | Strongly Disagree | Somewhat Disagree | Neutral | Somewhat Agree | Strongly Agree |
| I prefer to use the VirTeasy simulator without the VR headset. |  |  |  |  |  |
| I prefer to use the VirTeasy simulator with the VR headset. |  |  |  |  |  |
| I prefer not to use the VirTeasy simulator. |  |  |  |  |  |

**Regardless of your answer, why do you feel this way?**

Please write your response here:

**Participation in Further Studies**

We are looking for students to continue our study on the use of the VirTeasy simulator. Would you be willing to discuss this with us? *

Yes, I am willing.

No, I am not interested.

Thank you for participating in this survey!

Submit your questionnaire. Thank you for completing this questionnaire.

Table S5. Characteristics of the study population, inter-group comparison of haptic parameters (G1T1 vs. G2T1 and G1T2 vs. G2T2), and additional statistical analysis assessing the performance of students with prior VR experience when using the headset versus without it.

| **Population characteristics** | | **G1T1_non-immersive N=42** | **G1T2_immersive N=42** | **G2T1_immersive N=44** | **G2T2_non-immersive N=44** |
| --- | --- | --- | --- | --- | --- |
| N missing | | 2 | | 1 | |
| Age Mean years old ± SD (Min-Max) | | 20.75 ± 0.70 | | 20.85 ± 0.78 | |
| Previous experience with VR | | 3 | | 4 | |
| Sex Women (n/N) | | 31/43 | | *35/44* | |
|  | | | | | |
| **Haptic parameters** | | **G1T1_non-immersive N=43** | **G1T2_immersive N=43** | **G2T1_immersive N=44** | **G2T2_non-immersive N=44** |
| **Total time (min)** | Min-Max | [2.00;11.52] | [5.03;12.40] | [4.46;13.14] | [5.08;13.19] |
|  | Mean ± SD | 7.54 ±2.32 | 9.50 ±1.33 | 9.42 ±1.36a | 9.04 ±1.38a |
|  | Median [Q1;Q3] | 8.58 [6.07;10.12] | 10.20 [9.33;10.33] | 10.14 [8.51;10.31] | 9.20 [7.55;10.18] |
|  | p-value <0.05 | G1T1-G2T1  Mann-Whitney test | G1T2-G2T2  Student's t-test | G2T1-G2T1  Mann-Whitney test | G2T2-G1T2  Student's t-test |
| *Temps de chirurgie* | | | | | |
| **Drilling time (min)** | Min-Max | [1.27;8.09] | [2.25;9.33] | [2.53;9.19] | [2.49;8.43] |
|  | Mean ± SD | 4.54 ±1.40 | 5.52 ±1.46 | 5.57 ±1.28 | 5.31 ±1.30 |
|  | Median [Q1;Q3] | 4.50 [3.42;6.10] | 5.57 [4.28;7.18] | 5.49 [4.46;7.08] | 5.24 [4.24;6.47] |
|  | p-value <0.05 | */*  Student's t-test | G1T2-G2T2  Student's t-test | */*  Student's t-test | G2T2-G1T2  Student's t-test |
|  | | | | | |
| **Target progression (%)** | Min-Max | [64.4;93.8] | [49.3;99.6] | [58.1;95.0] | [57.2;96.0] |
|  | Mean ± SD | 88.2 ±6.3 | 82.8 ±11.3 | 84.6 ±8.5 | 87.5 ±8.7 |
|  | Median [Q1;Q3] | 90.5 [86.9;91.7] | 85.7 [77.7;90.6] | 88.5 [79.9;90.7] | 90.1 [87.6;92.1] |
|  | p-value <0.05 | G1T1-G2T1  Mann-Whitney test | G1T2-G2T2  Student's t-test | G2T1-G2T1  Mann-Whitney test | G2T2-G1T2  Student's t-test |
|  | | | | | |
| **Accuracy (%)** | Min-Max | [52.5;96.5] | [54.3;96.1] | [58.5;95.9] | [60.5;95.1] |
|  | Mean ± SD | 80.6 ±8.3 | 78.0 ±10.5 | 78.9 ±8.7 | 81.5 ±7.6 |
|  | Median [Q1;Q3] | 82.5 [75.8;86.7] | 80.2 [72.2;84.8] | 79.8 [72.2;85.4] | 82.1 [76.4;87.5] |
|  | p-value <0.05 | */*  Student's t-test | */*  Student's t-test | */*  Student's t-test | */*  Student's t-test |
|  | | | | | |
| **Inside volume**  **(IV, mm^3^)** | Min-Max | [128.3;187.0] | [98.4;198.6] | [115.8;189.5] | [114.1;191.5] |
|  | Mean ± SD | 175.9 ±12.7 | 165.0 ±22.6 | 168.7 ±16.9 | 174.5 ±17.3 |
|  | Median [Q1;Q3] | 180.3 [173.3;182.8] | 170.9 [154.9;180.7] | 176.5 [159.4;180.8] | 179.7 [174.6;183.5] |
|  | p-value <0.05 | G1T1-G2T1  Mann-Whitney test | G1T2-G2T2  Student's t-test | G2T1-G2T1  Mann-Whitney test | G2T2-G1T2  Student's t-test |
|  | | | | | |
| **Ouside volume**  **(IV, mm^3^)** | Min-Max | [5.4;150.8] | [4.0;151.8] | [4.9;116.4] | [8.4;109.5] |
|  | Mean ± SD | 45.6 ±27.4 | 53.0 ±37.5 | 48.8 ±27.1 | 42.0 ±22.4 |
|  | Median [Q1;Q3] | 36.1 [28.2;57.6] | 40.3 [27.7;69.5] | 43.6 [28.5;64.9] | 38.8 [25.5;55.4] |
|  | p-value <0.05 | */*  Mann-Whitney test | */*  Student's t-test | */*  Mann-Whitney test | */*  Student's t-test |
| **Additional analyses** | | | | | |
| **Haptic parameters** | | **G1T1_non-immersive previous experience with VR**  **N=3** | **G1T2_immersive**  **previous experience with VR**  **N=3** | **G2T1_immersive previous experience with VR**  **N=4** | **G2T2_non-immersive previous experience with VR**  **N=4** |
|  |  | Mean ± SD | Mean ± SD | Mean ± SD | Mean ± SD |
| **Total time (min)** | | 9.18 ±0.12* | 10.31 ±0.5* | 8.54 ±0.51^a^ | 7.11 ±0.14^b^ |
| **Drilling time (min)** | | 6.3 ±0.13* | 7.11 ±0.32* | 5.42 ±0.36^a^ | 4.03 ±0.45^b^ |
| **Target progression (%)** | | 90.87 ±0.76* | 85.53 ±2.76* | 90.03 ±1.21^a^ | 91.33 ±1.14^b^ |
| **Accuracy (%)** | | 83.5 ±4.27* | 68.5 ±9.47* | 72.73 ±3.39^a^ | 83.35 ±4.5^b^ |
| **Inside volume (IV, mm^3^)** | | 181.1 ±1.53* | 170.5 ±5.53* | 179.5 ±2.45^a^ | 182.08 ±2.21^b^ |
| **Ouside volume (IV, mm^3^)** | | 36.5 ±11.93* | 83.2 ±35.47* | 68.28 ±14.03^a^ | 37.15 ±12.25^b^ |

G1, group 1 ; T1, time 1 ; N, number of participants; T2, time 2; G2, group 2; min, minutes; Min, minimum; Max, maximum; SD, standard deviation; Q1, first quartile; Q3, third quartile; VR, virtual reality, * Significant difference (P < 0.05); /, non-significant difference between groups; G1T1-G2T1, G1T2-G2T2, G2T1-G2T1, G2T2-G1T2, significant difference between groups. For G2, different letters on the horizontal axis indicate statistically significant differences in haptic parameters between the two simulation conditions (P < 0.05).
